# Bond Strength of Sandblasted PEEK with Dental Methyl Methacrylate-Based Cement or Composite-Based Resin Cement

**DOI:** 10.3390/polym15081830

**Published:** 2023-04-09

**Authors:** Kentaro Hata, Yuya Komagata, Yuki Nagamatsu, Chihiro Masaki, Ryuji Hosokawa, Hiroshi Ikeda

**Affiliations:** 1 Division of Biomaterials, Department of Oral Functions, Kyushu Dental University, Kitakyushu 803-8580, Japan; 2 Division of Oral Reconstruction and Rehabilitation, Department of Oral Functions, Kyushu Dental University, Kitakyushu 803-8580, Japan

**Keywords:** dental adhesive, poly-ether-ether-ketone, methyl methacrylate, bond strength, airborne particle abrasion

## Abstract

Poly-ether-ether-ketone (PEEK) is commonly employed in dental prostheses owing to its excellent mechanical properties; however, it is limited by its low bond strength with dental resin cement. This study aimed to clarify the type of resin cement most suitable for bonding to PEEK: methyl methacrylate (MMA)-based resin cement or composite-based resin cement. For this purpose, two MMA-based resin cements (Super-Bond EX and MULTIBOND II) and five composite-based resin cements (Block HC Cem, RelyX Universal Resin Cement, G-CEM LinkForce, Panavia V5, and Multilink Automix) were used in combination with appropriate adhesive primers. A PEEK block (SHOFU PEEK) was initially cut, polished, and sandblasted with alumina. The sandblasted PEEK was then bonded to resin cement with adhesive primer according to the manufacturer’s instructions. The resulting specimens were immersed in water at 37 °C for 24 h, followed by thermocycling. Subsequently, the tensile bond strengths (TBSs) of the specimens were measured; the TBSs of the composite-based resin cements after thermocycling were found to be zero (G-CEM LinkForce, Panavia V5, and Multilink Automix), 0.03 ± 0.04 (RelyX Universal Resin Cement), or 1.6 ± 2.7 (Block HC Cem), whereas those of Super-Bond and MULTIBOND were 11.9 ± 2.6 and 4.8 ± 2.3 MPa, respectively. The results demonstrated that MMA-based resin cements exhibited stronger bonding to PEEK than composite-based resin cements.

## 1. Introduction

Poly-ether-ether-ketone (PEEK) is widely used as an engineering plastic in industry because of its superior mechanical strength, fatigue resistance, chemical durability, temperature resistance, and dimensional stability under harsh conditions. In the dental field, owing to its excellent biocompatibility and outstanding properties [1], PEEK has received significant research interest for use in dental implants [2], implant abutments [3], removable dentures [4], crowns [5], posts and cores [6], prostheses [7], orthodontic wires [8], and occlusal splints [9]. 

Despite its desirable properties, the appearance of PEEK is significantly different from that of natural teeth owing to its opacity and greyish pearly white color. When preparing a PEEK prosthesis, the color of the PEEK must be shielded by veneering with resin-based adhesive, which requires the use of adhesives. In fixed dental prostheses, PEEK is bonded onto the abutment teeth using an adhesive, which means that durable bonding between PEEK and the adhesive is indispensable to ensure that the prosthesis is stable over numerous years. However, the chemically inert nature and low surface energy of PEEK render adhesive bonding difficult. Thus, various surface-modification methods have been investigated to overcome these problems [8]. For example, alumina sandblasting (alumina airborne particle abrasion) can mechanically roughen the PEEK surface to increase the surface area, resulting in an improved bond strength [10,11,12]. 

Furthermore, plasma treatment using helium, argon, nitrogen, and oxygen gases can remove thin layers of the PEEK substrate, which also removes contaminants and modifies the functional groups of the PEEK surface to increase its wettability [13,14,15]. A laser abrasion induces microgrooves on the PEEK surface to ensure the mechanical retention of luting agents [16,17,18]. Acid etching causes surface topographical changes and provides a microinterlocking structure on the surface [6,19,20]. For example, etching with 98% sulfuric acid can create a porous surface structure wherein an adhesive penetrates, leading to significantly improved bond strength [21,22,23]. Overall, there is a consensus that alumina sandblasting and sulfuric acid etching are effective surface pretreatment methods for improving PEEK adhesion [24]. However, the use of such a high concentration of sulfuric acid is undesirable due to its toxicity and the risks associated with damage to the oral tissue [25,26]. Thus, considering its safety, versatility, and usability, alumina sandblasting is considered the most suitable technique for the surface pretreatment of PEEK.

In addition to the use of chemical, physical, and mechanical approaches to increase the bond strength between PEEK and an adhesive, the adhesive system itself is partly responsible for determining the resulting bond strength. For instance, the combination of an adhesive primer and resin cement has been demonstrated as a reliable adhesive system for bonding PEEK [6,27], wherein methyl methacrylate (MMA)-containing adhesive primers are particularly effective for bonding PEEK to resin cement [27,28]. Previous studies have reported that two MMA-containing primers, namely Visio.link and Signum PEEK Bond, can improve the bond strength of PEEK [6,29,30]. More specifically, alumina sandblasting followed by the application of an MMA-containing primer significantly increases the bond strength between PEEK and resin cement [12]. Similarly, alumina sandblasting and the application of an MMA-containing primer significantly improve the adhesion of PEEK crowns [31]. Furthermore, several resin cements have been investigated to improve the bond strength with PEEK [24], although the optimal resin cement system has yet to be identified.

The purpose of this study is to clarify the bonding performance of dental resin cement to sandblasted PEEK. Thus, the tensile bond strength (TBS) is investigated using two MMA-based resin cements and five composite-based resin cements. The null hypothesis is that there is no difference between the TBSs of MMA-based resin cements and composite-based resin cements in the context of PEEK bonding.

## 2. Materials and Methods

### 2.1. Materials

Details regarding the PEEK blocks, resin cements, and primers used in this study are listed in Table 1. The PEEK block was cut to a thickness of 4 mm using a diamond wheel saw and was polished using emery paper #600. The polished PEEK surface was sandblasted at 0.2 MPa with 50 μm alumina particles using an airborne particle abrader (Jet Blast II, J. Morita, Suita, Japan). The surface morphology of the PEEK was observed using scanning electron microscopy (SEM; S-4300, Hitachi High-Tech, Tokyo, Japan). The obtained sandblasted PEEK was used for all subsequent experiments.

### 2.2. Tensile Bond Strength (TBS) Test

**Figure 1:**
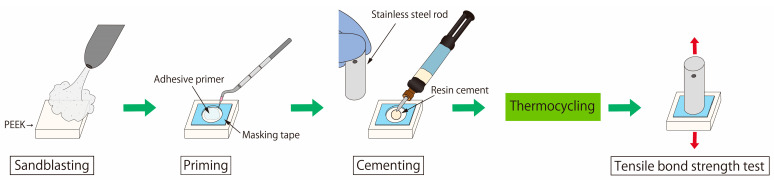
Flowchart of tensile bond strength test.

A flowchart of the tensile bond strength test is shown in Figure 1. For bonding with the sandblasted PEEK, each resin cement was used in combination with the adhesive primer provided by the same manufacturer, as listed in Table 2. Initially, the PEEK surface was covered with masking tape containing a 4.8 mm diameter hole to regulate the bonding area. The adhesive primer was applied to the masked PEEK surface according to the manufacturer’s instructions. The resin cement was then loaded onto the primed PEEK surface and bonded to an alumina-sandblasted stainless-steel rod. The bonded specimen was maintained under ambient conditions at 25 °C for 30 min to cure the resin cement and was subsequently immersed in distilled water at 37 °C for 24 h. The specimen was subjected to a thermocycling process for 20,000 cycles, which was conducted by alternating immersion in water baths at temperatures of 5 and 55 °C with a dwell time of 20 s in each bath. The resulting specimens (n = 10 per group) were used to carry out the TBS tests. The TBS between the resin cement and the PEEK sample was measured by a conventional TBS test procedure [32] using a universal testing machine (AGS-H, Shimadzu., Kyoto, Japan) at a crosshead speed of 1 mm/min. The maximum force was measured when the cement/PEEK interface was debonded. The TBS was calculated by dividing the maximum force by the adhesive area. After the TBS test, each debonded PEEK surface was observed by SEM to determine its failure mode. The failure modes were classified into three types, namely, adhesive failure at the PEEK/cement interface, cohesive failure within the cement, and mixed adhesive and cohesive failures.

### 2.3. Statistical Analysis

The TBS data were analyzed using EZR statistical software (EZR version 1.61, Saitama Medical Center, Saitama, Japan). Two-way analysis of variance (ANOVA) was used to evaluate the significant influences of the resin cement and the thermocycling process. A subgroup analysis was also performed using Tukey’s test for multiple comparisons of the different resin cement groups. In addition, Student’s t-test was used to determine the effect of thermocycling on each group. The significance level was set to *p* < 0.05 for all results.

## 3. Results

### Preparation of the Alumina-Sandblasted PEEK

**Figure 2:**
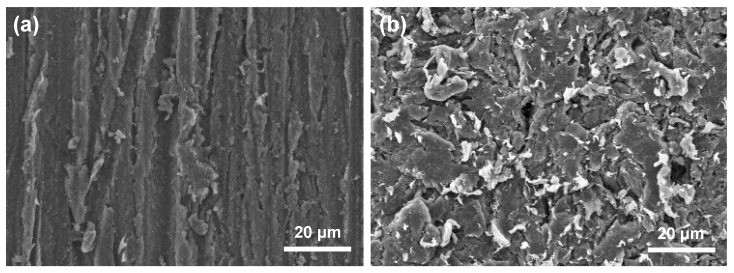
SEM images for the PEEK surfaces: (**a**) surface polished using emery paper and (**b**) alumina-sandblasted surface.

Alumina sandblasting was employed to roughen the surface of the PEEK and increase the surface area available for adhesive bonding. As shown in Figure 2, numerous microgrooves were introduced into the surface structure via sandblasting.

Table 3 lists the results of the two-way ANOVA carried out for the obtained TBS results. As indicated, both factors, namely, the “resin cement” and the “thermocycling” factors, had statistically significant effects on the TBS (*p* < 0.001); the interaction between these factors was also statistically significant (*p* < 0.001).

**Figure 3:**
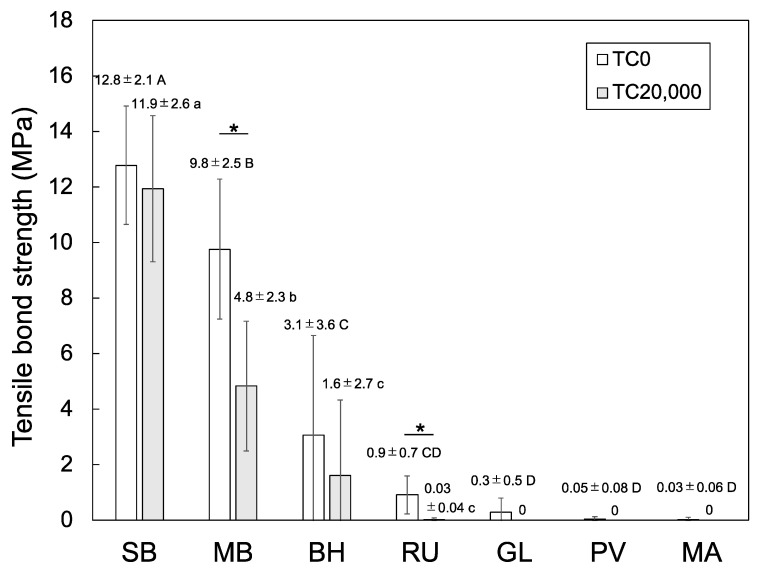
Mean and standard deviations of the TBSs of the various experimental groups. Different uppercase and lowercase letters represent statistically significant differences among the non-thermocycled (TC0) and thermocycled (TC20,000) groups, respectively. An asterisk indicates a statistical difference between the TBSs for the TC0 and TC20,000 groups for the same resin cement.

As can be seen from Figure 3, the TBS was clearly influenced by the type of resin cement employed. Upon comparison among the non-thermocycled groups (TC0), the TBSs obtained for the MMA-based resin cements (SB and MB) were significantly higher than those for the composite-based resin cements (BH, RU, GL, PV, and MA). Following thermocycling (TC20,000), the TBSs of the composite-based resin cements of the GL, PV, and MA groups were zero, suggesting that the bond strengths were extremely low, resulting in debonding of the cements during thermocycling. For the MMA-based resin cements (SB and MB), the TBS obtained for the SB group after thermocycling was significantly higher than that of the MB group. In addition, the TBS obtained for the SB group was found to remain constant after thermocycling, whereas that of the MB group decreased significantly.

The failure modes of the various groups were then determined, and are listed in Table 4. For the composite-based resin cements (RU, GL, PV, and MA), only adhesive failure was observed in all samples, with the exception of the BH group. In contrast, mixed failures were observed for the MMA-based resin cements (SB and MB). These results imply that the MMA-based resin cements bonded more strongly to PEEK than the composite-based resin cements.

## 4. Discussion

Contemporary resin cements can be classified into two groups, MMA-based and composite-based, according to their material composition. The main components of the former resin cement are MMA and PMMA, and the composite-based resin cement is made of a composite of a resin matrix and ceramic particles (fillers). Due to the differences in their compositions, the bonding properties of the MMA-based and composite-based resin cements as restorative materials are different. This study aimed to reveal the bonding behaviors of the resin cements to PEEK. The TBS tests were conducted using two commercial MMA-based resin cements and five composite-based resin cements to determine the type of adhesive system that exhibited the strongest bonding with PEEK. The obtained results revealed that the TBSs of the MMA-based resin cements were significantly higher than those of the composite-based resin cements. Therefore, the null hypothesis that there is no difference between the TBSs of MMA-based resin cements and composite-based resin cements in PEEK bonding was rejected.

To estimate the bonding durability, the specimens were subjected to 20,000 thermocycles. These conditions imitate the temperature fluctuations that occur in the oral cavity, corresponding to an in vivo time of 2 years [33]. During thermocycling, water penetrates the cement/PEEK interface and deteriorates the bonding via hydrolysis. As a result, almost all resin cement groups showed a reduced TBS after thermocycling. More specifically, in the composite-based resin cement groups, the TBSs decreased to approximately zero following thermocycling, implying that the bonding of the resin cement to PEEK would be lost within 2 years in the oral environment. Furthermore, some samples from the RU, GL, PV, and MA groups exhibited debonding during thermocycling, suggesting that composite-based resin cements debond from PEEK in oral environments within a short period of time.

Adhesive primers are known to play an important role in enhancing the bond strengths between restorative materials and resin cements. In the case of commercial adhesive primers, these materials contain organic solvents and several functional monomers that chemically react with both the resin cement and restorative material surface [34]. The adhesive primers used in the current study contain one or more of the following functional monomers: MDP, γ-MPTS, 4-META, MTU-6, or VTD (VBATDT). Notably, each of these functional monomers can adhere to a specific material surface depending on the chemical structure. More specifically, MDP binds preferentially to zirconia and titanium [35], γ-MPTS binds to silica-based glass ceramics and silica-containing resin composites [36], 4-META is preferred for base metal alloys [37], and MTU-6 and VTD bind preferentially to noble metals [38]. However, these functional monomers are considered ineffective for PEEK bonding, as confirmed by the obtained experimental results, wherein the TBSs of the composite-based adhesive systems were close to zero (Figure 3); adhesion of the functional monomers to the PEEK surface would have led to increased TBSs. As PEEK is an aromatic, semi-crystalline, and chemically stable linear thermoplastic polymer [39], it contains no surface sites that can react with the functional monomers present in the adhesive primer. Indeed, no effective functional monomers have been reported for combination with PEEK in dental applications; this study was also unable to identify an effective functional monomer for PEEK bonding. Systematic experiments are therefore required to identify appropriate functional monomers for PEEK bonding to resin cement.

The TBS results suggested that the MMA-based resin cements provided better bonding to the sandblasted PEEK than the composite-based resin cements. Two possible explanations were considered for this observation. First, it was considered that a semi-interpenetrating polymer network (semi-IPN) structure [40,41,42,43] formed at the cement/PEEK interface. This semi-IPN structure consists of a macromolecular-level polymer blend, in which the polymer chains of the linear polymer penetrate another polymer network. In dental materials, a semi-IPN structure can be found at the interfaces between PMMA-based and resin-based materials, which significantly improves the bond strength between the two materials [40,41,42,43]. It was therefore speculated that the relatively small MMA molecules present in the MMA-based resin cements can penetrate the interspaces between the polymer chains, resulting in interlocking between the polymers, generating PMMA and forming a semi-IPN structure at the interface. This IPN structure contributes to enhancing the TBS between the MMA-based resin cement and PEEK. In contrast, composite-based resin cements typically contain relatively large molecules, such as TEGDMA and UDMA, and as a result, they are generally unable to penetrate the spaces between the PEEK molecules, and an IPN structure cannot be formed. In terms of the second possible explanation for the described observations, the wettability of the resin cement can be considered. As the sandblasted PEEK surfaces contain numerous grooves of various depths and sizes (Figure 2), the relatively low viscosity of the MMA-based resin cement allows it to infiltrate the narrow grooves on the PEEK surface. The penetrated MMA can then become cured to form PMMA, leading to mechanical interlocking at the cement/PEEK interface. In contrast, composite-based resin cements contain numerous ceramic fillers (particles), and these fillers are unable to infiltrate the narrow grooves on the PEEK surface, and so mechanical interlocking is unlikely to occur at the composite-based resin cement/PEEK interface.

Based on the results of the above in vitro experiments, MMA-based resin cements are apparently superior for bonding to alumina-sandblasted PEEK. However, even when the MMA-based resin cements are employed, the bond strength toward PEEK may degrade over time in the oral environment. Indeed, the actual oral environment is considered to be harsher than that simulated by the current in vitro conditions, due to the presence of occlusal forces, pH fluctuations, and other unfavorable factors. Further clinical studies are therefore required to clarify the effectiveness of MMA-based resin cements for PEEK bonding.

## 5. Conclusions

The bond strength between dental MMA-based resin cement or composite-based resin cement and the sandblasted PEEK was investigated by means of a tensile bond strength test, to determine the superior resin cement for durable bonding. The TBSs of the composite-based resin cements after thermocycling were found to be ≤2 MPa, whereas those of MMA-based resin cements (Super-Bond and MULTIBOND) were 11.9 ± 2.6 and 4.8 ± 2.3 MPa, respectively. The tensile bond strengths for the MMA-based resin cement were significantly higher than those of the composite-based resin cements. Within the limitations of the study, it was found that MMA-based resin cements are suitable to bond with PEEK for dental restoration.

## Figures and Tables

**Table 1:** Materials used in the current study. The material compositions are based on the information provided by the respective manufacturers.

Material Type	Product Name	Manufacturer	Composition
PEEK block	SHOFU PEEK	Shofu, Kyoto, Japan	Poly-ether-ether-ketone
MMA-based resin cement	Super-Bond EX *	Sunmedical, Moriyama, Japa	MMA, PMMA, 4-META, TBB-O
	MULTIBOND II	Tokuyama Dental, Tokyo, Japan	PMMA, co-activator, MMA, UDMA, HEMA, MTU-6, borate catalyst.
Composite-based resin cement	Block HC Cem	Shofu, Kyoto, Japan	UDMA, TEGDMA, silica powder, fine particulate silica, zirconium silicate, colorant.
	RelyX Universal Resin Cement	3M, Saint Paul, USA	Methacrylate, silica, glass powder, co-activator.
	G-CEM LinkForce	GC, Tokyo, Japan	UDMA, dimethacrylate, stabilizer.
	Panavia V5	Kuraray Noritake Dental, Tokyo, Japan	Bis-GMA, TEGDMA, titanium dioxide.
	Multilink Automix	Ivoclar Vivadent, Schaan, Liechtenstein	Ytterbium trifluoride, ethyoxylated Bis-GMA, Bis-GMA, HEMA, 2-dimethylaminoethyl methacrylate.
Adhesive primer	M&C Primer	Sunmedical, Moriyama, Japan	Primer A: MDP, VTD, MMA, acetone; Primer B: γ-MPTS, MMA.
	BONDMER Lightless	Tokuyama Dental, Tokyo, Japan	Primer A: MDP, MTU-6, Bis-GMA, TEGDMA, HEMA, acetone; Primer B: γ-MPTS, isopropanol, water, initiators, acetone.
	MULTIBOND II Primer	Tokuyama Dental, Tokyo, Japan	Phosphoric acid monomer, water, acetone, UDMA, co-activator.
	HC Primer	Shofu, Kyoto, Japan	UDMA, MMA, photo-initiator, acetone, and others.
	Scotchbond Universal Plus Adhesive	3M, Saint Paul, USA	Phosphoric acid ester monomer, methacrylate, co-activator, ethanol.
	G-Multi PRIMER	GC, Tokyo, Japa	Ethanol, phosphoric acid ester monomer, dimethacrylate component.
	CERAMIC PRIMER PLUS	Kurary Noritake Dental, Tokyo, Japan	Ethanol, γ-MPTS, MDP.
	Monobond Plus	Ivoclar Vivadent, Schaan, Liechtenstein	Ethanol, methacrylated phosphoric acid ester.

MMA: methyl methacrylate, 4-MEMA: 4-methacryloxyethyl trimellitic anhydride, UDMA: urethane dimethacrylate, TBB-O: partially oxidized tri-*n*-butyl borane, Bis-GMA: bisphenol A diglycidyl-methacrylate, TEGDMA: triethyleneglycol dimethacrylate, HEMA: 2-hydroxyethyl methacrylate, MDP: 10-methacryloyloxydecyl dihydrogen phosphate, γ-MPTS: 3-methacryloxypropyl trimethoxy silane, VTD (VBATDT): 6-(4-vinylbenzyl-*n*-propyl)amino-1,3,5-triazine-2,4-dithione. * Trade name in Europe: Super-Bond Universal.

**Table 2:** Combinations of the resin cements and primers used for PEEK bonding.

Adhesive System	Resin Cement	Primer	Group
MMA-based resin cement	Super-Bond EX	M&C Primer	SB
	MULTIBOND II	BONDMER Lightless and MULTIBOND II primer	MB
Composite-based resin cement	Block HC Cem	HC Primer	BH
	RelyX Universal Resin Cement	Scotchbond Universal Plus Adhesive	RU
	G-CEM LinkForce	G-Multi PRIMER	GL
	Panavia V5	CERAMIC PRIMER PLUS	PV
	Multilink Automix	Monobond Plus	MA

**Table 3:** Results of the two-way ANOVA carried out for the TBS results between PEEK and each resin cement, both with and without thermocycling.

	Sum of Square	*Df*	*F* Value	*p* Value
Resin cement	2767.59	6	146.5685	<0.001
Thermocycling	51.36	1	16.3213	<0.001
Resin cement * thermocycling	88.60	6	4.6921	<0.001

**Table 4:** Failure modes of the various experimental groups in the TBS tests.

	Adhesive/Mixed/Cohesive *	
Group	TC0	TC20,000
SB	2/8/0	0/10/0
MB	9/1/0	6/4/0
BH	8/2/0	8/2/0
RU	10/0/0	10/0/0
GL	10/0/0	10/0/0
PV	10/0/0	10/0/0
MA	10/0/0	10/0/0

* Adhesive: adhesive failure at the PEEK–cement interface. Cohesive: cohesive failure within cement. Mixed: mixed failure of adhesive and cohesive.

## Data Availability

The data presented in this study are available upon request from the corresponding author.
